# Randomization across breeding cohorts improves the accuracy of conventional and genomic selection

**DOI:** 10.1002/tpg2.70218

**Published:** 2026-03-16

**Authors:** Arlyn Ackerman, Jessica Rutkoski

**Affiliations:** ^1^ Breeding Insight Cornell University Ithaca New York USA; ^2^ Department of Crop Sciences University of Illinois at Urbana‐Champaign Champaign Illinois USA

## Abstract

Breeding programs conventionally evaluate cohorts in separate trials; however, environmental differences across testing areas can be confounded with genetic differences between cohorts, potentially reducing the accuracy of breeding value estimation. We test whether the conventional approach of restricting randomization of cohorts to *within* trials reduces genomic and conventional selection accuracy when compared to the complete randomization of all cohorts *across* a trial, using in silico simulation with marker data from University of Illinois winter wheat breeding lines. We evaluated selection accuracy for conventional best linear unbiased prediction (BLUP), genomic BLUP (GBLUP), and genomic‐enabled sparse testing across a comprehensive simulation space spanning narrow‐sense heritabilities of 0.2–0.8, genetic correlations between testing areas from 0.2 to 1.0, and three replication levels. Difference‐in‐differences (DiD) analysis established causal inference by comparing design performance as conditions deteriorated from an optimal baseline where both designs performed equivalently. Complete randomization improved BLUP accuracy by 11.7%, reaching 15.7% under low replication and low genetic correlation between areas. Genomic data largely eliminated this design effect, with GBLUP showing no significant DiD interaction effect. However, genomic‐enabled sparse testing revealed a significant DiD effect and an improvement in selection accuracy of 1.5% that increased to a 5.5% advantage under challenging conditions. While heritability had the strongest main effect on selection accuracy, genetic correlation between areas showed the largest interaction with randomization scheme, with design performance diverging significantly only as this parameter decreased. Programs with genomic data and balanced phenotypic data can use either restricted or complete randomization, but those with other circumstances can benefit from complete randomization.

## Plain Language Summary

In breeding programs, groups of related breeding materials, called cohorts, are often tested in separate trials. As a result, confounding of genetic and non‐genetic effects occurs. This confounding is expected to reduce the accuracy of selection when cohorts are analyzed together for selection or predictive modeling. We evaluated the extent to which randomizing all cohorts together for testing improves selection accuracy relative to randomization solely within cohorts. To do this, we simulated a range of scenarios and assumptions and determined the accuracy gained from complete randomization across cohorts in each case. We found that randomization across cohorts provided large benefits when genomic relationship information was absent and modest benefits when data were unbalanced but genomic relationship information was present. Breeding programs should strongly consider randomizing cohorts together whenever feasible.

**Abbreviations**
BLUP: best linear unbiased prediction
CR: complete randomization
DiD: difference‐in‐differences
G × E: genotype‐by‐environment interaction
GBLUP: genomic best linear unbiased prediction
GRM: genomic relationship matrix
GS: genomic selection
RR: restricted randomization

## 1 INTRODUCTION

Yield trials are a cornerstone of breeding programs, essential for achieving genetic gain through selection, but often requiring significant resources. Yield trials are conducted to generate phenotypic data on breeding materials, such as lines, families, and hybrids, to support selection decisions and train predictive statistical models. However, phenotypes are a culmination of both genetic and non‐genetic factors, and in the case of yield, most of the phenotypic variation is non‐genetic. Plant breeders who make selection decisions based on total genetic values or breeding values therefore must employ statistical designs appropriate for yield trials to facilitate the separation of genetic from non‐genetic effects. To ensure accurate estimates of genetic or breeding values, replication within and across environments is crucial, as is blocking and proper research plot technique. We define a single yield trial as one that takes place in a unique location‐year combination and is developed using a single randomization process and entry list. In breeding programs, multiple yield trials are often conducted across environments as multi‐environment yield trials and ultimately analyzed together to obtain more accurate estimates of genetic or breeding values.

To adequately sample environments, yield testing is conducted across years, in which only the best 15%–30% of breeding materials will be advanced for testing the following year. To accommodate the selection and advancement of breeding materials through yield trials, breeding programs utilize formalized multi‐environment testing processes. A consistent process is applied to each cohort of breeding materials that enters multi‐location testing. A cohort is defined as a set of genotypes within a specific selection stage of the advancement process (Covarrubias‐Pazaran et al., 2022). In yield trials, cohorts are often referred to by their stage of testing or by their selection cycles (Cullis et al., 2020). For example, genotypes in their first year of yield testing may be referred to as stage one (S1). Genotypes in their second, third, and fourth years of yield testing may be referred to as stage two (S2), stage three (S3), and stage four (S4), respectively (A. Smith et al., 2021). Genotypes belonging to the same cohort are typically generated from the same crossing block; therefore, they are expected to be more closely related to each other compared to genotypes from different cohorts. Typically, cohorts are evaluated in separate yield trials; however, two or more cohorts can sometimes be evaluated in the same trial. One common testing approach is to evaluate S1 genotypes together in what is known as an observation yield trial, S2 genotypes together in what is known as a preliminary yield trial, and finally, S3 and S4 genotypes together in what is known as an advanced yield trial. Another common practice is to evaluate S1 and S2 genotypes together within a preliminary yield trial, followed by an advanced yield trial. Regardless, separating cohorts into different trials effectively restricts randomization to within groups of genotypes that are more closely related.

For each individual yield trial, design factors such as the replication per entry and the proportion of genotypes selected for further testing generally remain consistent from year to year within the breeding program. The replication of test entries scales with advancement through the trial stages: entries in observation trials are typically unreplicated, those in preliminary yield trials may be unreplicated or replicated twice, and advanced yield trials generally employ two or three replicates (Bernardo, 2010). Selection for advancement to the next stage is performed within a cohort, and evaluating cohorts in separate trials is intended to improve this within‐cohort selection accuracy.

In addition to generating data used to make advancement decisions, yield trials often serve as a source of phenotypic data for training genomic prediction models to facilitate genomic selection (GS). As reviewed by Lorenz and Nice (2017), GS has revolutionized selection in breeding programs. In the context of GS, a genomic prediction model is trained with genotypic and phenotypic data and then used for selection (Jannink et al., 2010). This enables the selection of parents among genotypes that have not yet been tested extensively or at all, thereby enabling shorter breeding cycles. Training population sizes and genomic relationships between test and training sets are critical for prediction accuracy (Combs & Bernardo, 2013; Lorenz et al., 2011; Lorenz & Smith, 2015). The implementation of GS also facilitates “sparse testing” of entries across a larger number of environments (Atanda et al., 2021; Heffner et al., 2010; Verges & Sanford, 2020). In the case of sparse testing, breeding materials are intentionally evaluated in different sets of environments and analyzed together in a GS model that enables the prediction of breeding values despite the unbalanced testing design.

In most GS use cases, breeders must routinely combine data from different cohorts for analysis to train and update GS models. Data from yield trials is used for GS model training in many breeding programs because it contains many unique genotypes with high‐quality data from multiple environments (Combs & Bernardo, 2013; Lorenz & Smith, 2015). Each year, new data from yield trials is generated, facilitating the updating of GS models required to maintain accuracy (Bassi et al., 2016; Gaynor et al., 2017). The advent of GS has increased the need for effective analysis across cohorts to train accurate prediction models.

A potential problem arises when using yield trial data to train GS models if cohorts are tested in separate yield trials. Genetic differences between cohorts and non‐genetic differences between trials may be confounding (Clarke & Stefanova, 2011; Robbins et al., 2012). Furthermore, genotype‐by‐trial interaction may be present and poorly separated from other effects. These problems could potentially reduce GS model accuracy. While studies have not examined how restricting randomization to within cohort impacts GS accuracy, a study by Piepho and Williams (2006) examined the efficiency of complete randomization (CR) across all breeding materials with that of restricted randomization (RR), in which randomization was performed within groups of closely related breeding materials. The authors ultimately concluded that if selection occurs within groups, RR should be used; if selection occurs across groups, CR should be used.

The use of common entries across trials, referred to as checks, can help mitigate confounding between genetic and non‐genetic factors in testing designs and improve accuracy; however, there are costs associated with check plots. We refer to two types of check entries: design checks and performance checks. Design checks are the entries included in the statistical design solely to facilitate the estimation of genetic and non‐genetic effects as well as variances. When analyzing multiple trials together, multiple design checks must be shared between trials to estimate the trial effects appropriately. In addition, design checks are needed to estimate block effects and residual error variances in trials designed to evaluate unreplicated test entries. Design checks may occupy up to 20% of available plots in breeding trials (Chandra, 1994; Martin et al., 2006; Moehring et al., 2014), or as suggested by Fisher (1926), the square root of the number of test plots. This is a considerable expense that breeding programs must offset by testing fewer lines. In addition to design checks, performance checks represent a market variety and product profile that is to be replaced, acting as a benchmark to which test lines are compared (Cobb et al., 2019). Occasionally, performance checks will be used as design checks to save resources; however, complete reliance on performance checks for the estimation of error variances, block effects, and trial effects can result in poor estimation of genetic effects for unreplicated lines if performance checks are not representative of the test entries (Moehring et al., 2014).

Rather than conduct separate trials for lines in different stages of testing and rely on design checks to ensure block and trial effects are estimated accurately, lines in different stages of testing could be randomized together. This approach could limit the confounding of genetic and non‐genetic factors while maximizing the number of plots allocated to selection candidates. The breeding program's selection and advancement procedures could remain unchanged. If all cohorts are to be replicated, combining different cohorts together in the same trial can be achieved with familiar designs such as RCBD or incomplete block designs. Typically, in a breeding program, at least one cohort will not be replicated, in which case partially replicated designs, or *p*‐reps, first proposed by Cullis et al. (2006), are useful. In a *p‐*rep, test entries that are at later stages of testing can serve as the replicated entries, enabling the estimation of block effects and the error variance. An integral aspect of *p*‐reps is that the replicated entries are randomized together with unreplicated entries in a single design to avoid design checks, thereby maximizing the number of test plots for a given budget. Importantly, the percentage of plots that are replicated test plots should be at least 20% to estimate the error variance and block effects effectively. Clarke and Stefanova (2011) found that the increased number of replicated test plots replacing check plots within a *p*‐rep design increased error degrees of freedom (df) and average precision.

Prior research has not explicitly examined how restricting randomization to within cohorts affects selection accuracy. Statistical design principles and a previous study by Piepho and Williams (2006) led us to hypothesize that CR may help improve the accuracy of selection based on breeding value estimates resulting from a combined analysis across trials. Hence, the overarching goal of this research is to test the hypothesis that switching from RR *within* breeding cohorts to CR *across* breeding cohorts improves conventional and GS accuracy when selection is performed across cohorts.

## 2 MATERIALS AND METHODS

To test our hypothesis, we simulated breeding program scenarios replicating CR and RR conditions. The following overview outlines our approach before detailing specific methods in subsequent subsections. We first establish key terminology relevant to the central question, then detail the simulation of breeding values and phenotypes, and finally describe the statistical models used to estimate selection accuracy.

**FIGURE 1:**
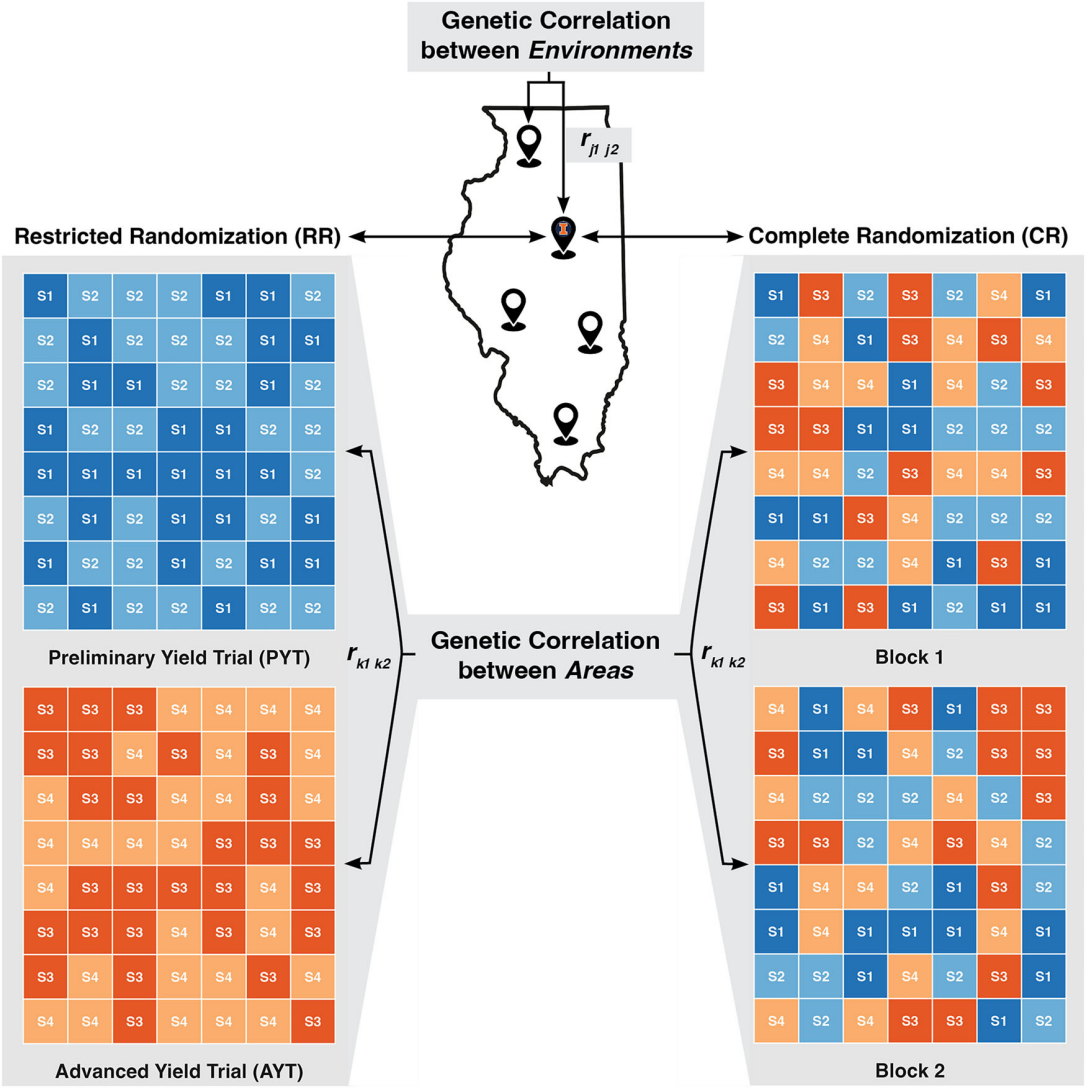
Depiction of the testing designs and hierarchy of genetic correlations simulated. A whole environment is represented with a grey rectangle, while areas within environments are represented by the two colored rectangles within each grey rectangle. Five environments were simulated with varying levels of genetic correlation between environments, $r_{j_{1}j_{2}}$. Under restricted randomization (RR), the areas within environment represent two separate trials with preliminary cohorts, S1 and S2, randomized separately from advanced cohorts, S3 and S4. Under complete randomization (CR), areas within environment represent blocks of a single trial with preliminary and advanced cohorts randomized together. Five levels of genetic correlation between areas were evaluated ($r_{k_{1}k_{2}}$ **= 1.0, 0.8, 0.6, 0.4, or 0.2). Lower $r_{k_{1}k_{2}}$ values indicate stronger genotype‐by‐area interaction, which may occur due to spatial separation or differential management of the areas.

**FIGURE 2:**
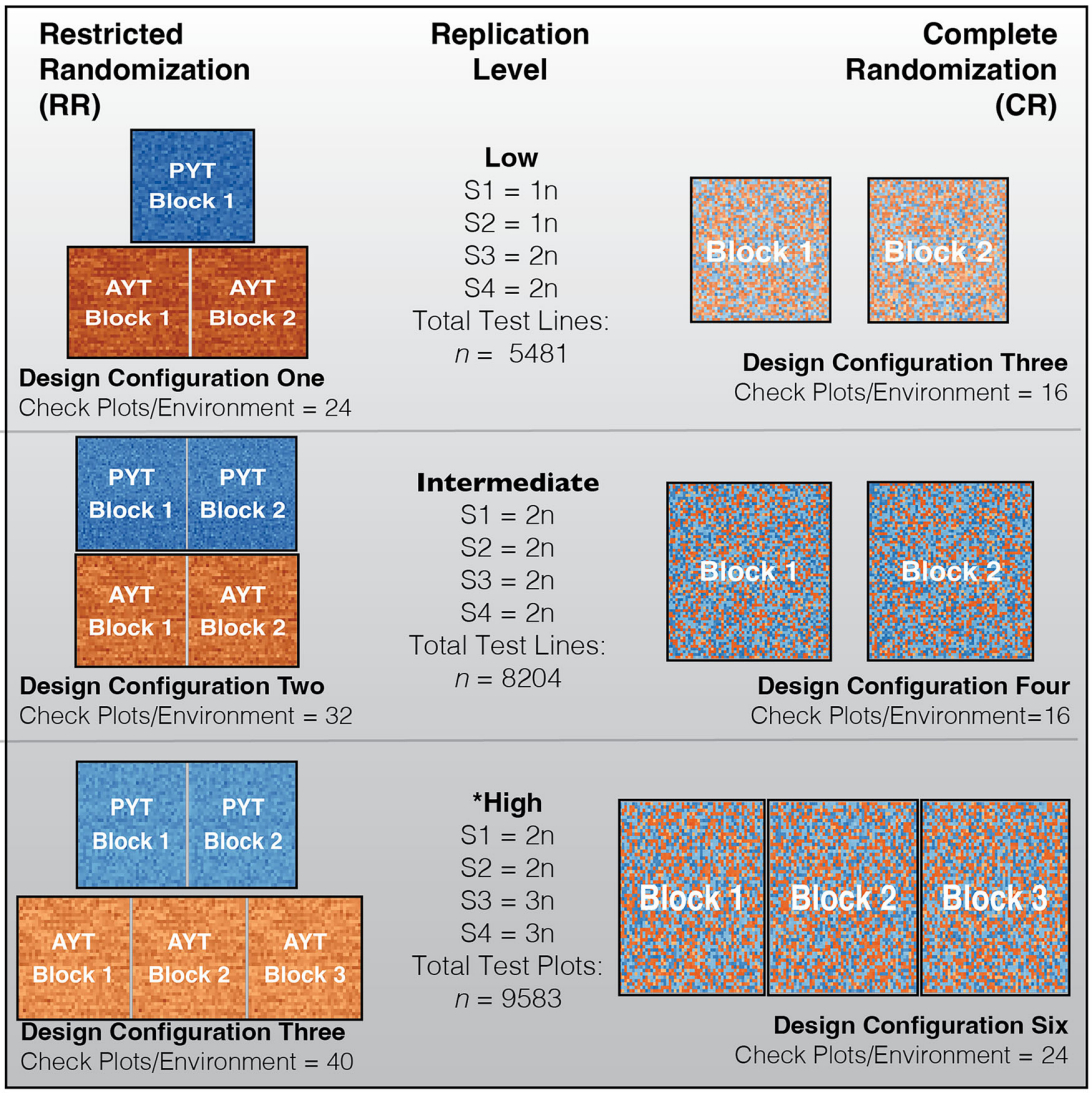
Corresponding design configurations for each replication level. Design configurations are shown as heatmaps for a single simulation run. Cohorts are color‐coded with S1, S2, S3, and S4 corresponding to dark blue, light blue, dark orange, and light orange, respectively. Replication level, replication per cohort, total test plots per location, and total check plots per environment are displayed next to each design. Comparable pairs of design configurations are shown side by side, with restricted randomization (RR) shown on the left and complete randomization (CR) shown on the right. With RR, preliminary cohorts S1 and S2 are randomized together to simulate a preliminary yield trial (PYT) while advanced cohorts S3 and S4 are randomized together to simulate an advanced yield trial (AYT). Each pair shares constant test plot numbers, *n*, and maintain eight checks per block across all designs. Black boxes indicate a single area and pairwise genetic correlations ($r_{k_{1}k_{2}}$) were simulated between these areas. Note that in the high replication scenario, RR and CR had two and three different areas, respectively. In contrast, both RR and CR had equal numbers of areas in the intermediate and low replication scenarios.

Throughout this work, we define an *environment* as a unique location‐year combination (e.g., Urbana South Farm—2022). Within each environment, there exist spatially separate *areas* suitable for research plots (Figure 1). Areas are central to our comparison of two randomization strategies. Within this study, the term “area” describes sections within a single environment that may differ in soil characteristics, microclimate, or management timing—subdivisions that commonly serve as either trials or blocks depending on the experimental design. In RR, cohorts are allocated to different areas within an environment, with each area functioning as a distinct trial containing separate breeding cohorts (e.g., preliminary and advanced trials). In CR, all cohorts are randomized together as a single trial (e.g., *p*‐rep design), with areas serving as blocks within that trial. Whether an area exists as a trial in RR or a block in CR, the number of test plots per area and the number of areas per environment were held constant between pairwise comparisons of RR and CR to ensure experimental integrity (with one exception at high replication, as shown in Figure 2).

Phenotypes reflective of a highly quantitative trait similar to yield were generated to populate these experimental designs using actual marker data from University of Illinois winter wheat breeding lines. Marker effects were first simulated with additive genetic covariances modeled between areas within environments and between separate environments, creating genotype‐by‐environment interaction (G × E) at two spatial scales. From these correlated marker effects, additive genetic values were computed for each line within each area‐environment combination, and a single true breeding value was then calculated for each genotype as the mean across all area‐environment‐specific genetic values. Residual error was subsequently added to create phenotypes at designated narrow‐sense heritabilities (0.2–0.8). For each simulation scenario, we fitted conventional best linear unbiased prediction (BLUP), genomic BLUP (GBLUP), and genomic‐enabled sparse testing models to the simulated phenotypes. Selection accuracy was assessed as the Pearson correlation between predicted and true breeding values and compared between randomization schemes across gradients of heritability, genetic correlation between testing areas (0.2–1.0), and replication level.

### 2.1 Genetic materials and genotyping

The wheat breeding lines utilized in this study consisted of F_3_‐derived lines from four different breeding cohorts (Table 1) developed by the University of Illinois wheat breeding program. All four cohorts were evaluated for yield in 2022 in Illinois as part of the breeding program's standard evaluation process. Cohorts S1, S2, S3, and S4 were in their first, second, third, and fourth years of testing, respectively. Lines belonging to the same cohort were developed from the same set of parental lines and are expected to be slightly more genetically related than lines from different cohorts. Cohorts S1 and S2 were designated as preliminary, and S3 and S4 were designated as advanced (Table 1). Entire cohorts S1, S2, and S3 were included in this study. Cohort size varied due to variability in the number of breeding candidates developed each year. For cohort S4, only the lines that were advanced to the second year of testing were genotyped; thus, just 20% of the original S4 cohort was included in this study. All lines were genotyped using genotyping‐by‐sequencing performed by the Eastern Regional Small Grains Genotyping Lab as described by Gaire et al. (2021) and Gaire et al. (2022). In total, 12,505 SNPs with call rates of at least 20% were obtained and used when simulating breeding values. For analysis, markers with minor allele frequencies lower than 0.05 were excluded, resulting in 9262 SNP markers.

**TABLE 1:** Description of breeding cohorts.

Cohorts	Number of lines	Designation
Stage 1	1204	Preliminary
Stage 2	1519	Preliminary
Stage 3	1151	Advanced
Stage 4	228	Advanced

### 2.2 Assessment of population structure

Using the marker data on all lines, we computed a genomic relationship matrix (GRM) calculated according to the additive relationship approach of Endelman (2011), as implemented by the *A.mat* function from the *rrBLUP* package. A principal component analysis (PCA) was performed on the GRM using the package ASRgenomics (Gezan et al., 2022). We then examined the proportion of the variance explained by the first two principal components and created a scatter plot of the first two principal components to visualize population structure.

### 2.3 Simulating marker effects and breeding values

For each area within an environment, we assumed that markers had different but correlated effects to create genotype‐by‐area interaction. To accomplish this, marker effects were sampled from a multivariate normal distribution with genetic covariances between areas, simulated according to the covariance matrix **R**. Here, we illustrate **R** for the case of two environments and two areas within each environment:

(1) $$R=\left[\begin{array}{cccc} 1 & \sigma_{k_{12}} & 0.5 & 0.5\\ \sigma_{k_{21}} & 1 & 0.5 & 0.5\\ 0.5 & 0.5 & 1 & \sigma_{k_{12}}\\ 0.5 & 0.5 & \sigma_{k_{21}} & 1 \end{array}\right]\;$$

Matrix **R** is the covariance between each area within an environment, with ones along the diagonal corresponding to the genetic variances simulated. The genetic covariance between environment one, area one and environment one, area two is $\sigma_{k_{12}}$. The genetic covariance between environment one, area one, and environment two, area one is 0.5. The genetic covariance between environment one, area one and environment two, area two is 0.5. The genetic covariance between environment one, area two and environment two, area one is $\sigma_{k_{21}}$. The genetic covariance between environment one, area two and environment two, area two is 0.5. The genetic covariance between environment two, area one and environment two, area two is $\sigma_{k_{12}}$. The notation $\sigma_{k_{12}}$ denotes the genetic covariance between areas *k*_1_ and *k*_2_ within an environment, defined as the genetic variance of $\sigma_{g}^{2}\cdot r_{k_{1}k_{2}}$, where $\sigma_{g}^{2}$ is the genetic variance and $r_{k_{1}k_{2}}$ is the genetic correlation between those areas. The factor $r_{k_{1}k_{2}}$ was manipulated in our simulation to control the magnitude of genotype‐by‐area interaction.

In our simulation, there were five environments with two areas in each, thus **R** was a 10 × 10 matrix. Correlated marker effects across *p = *10 areas within environments were simulated using the R function *mvrnorm* from package “MASS” (Venables & Ripley, 2002) using the matrix **R** as the covariance matrix. Each marker had *p* different effects corresponding to each area within an environment, and marker effects were simulated for each SNP.

Additive genetic values for each of the 4106 lines within each area within environment were generated from simulated marker effects by solving for the product of genotype matrix **M** of order *m × n* and the marker effect matrix **Q** of order *n × p*. For each line, a single true breeding value was then computed as the average across all 12 additive genetic values. Because all SNP markers were assigned an effect, the trait we simulated was highly quantitative, like yield.

### 2.4 Simulating phenotypes

To simulate phenotypic values from the environment and area‐specific additive genetic values for our breeding lines, we defined phenotypic value as follows:

(2) $$y_{ijk}=g_{i\left(jk\right)}+e_{j}+\varepsilon_{ijk}$$

where $y_{ijk}$ is the phenotypic value of genotype *i* within environment *j* and area *k*, $g_{i(jk)}$ is the additive genetic value of line *i* in environment *j* and area *k*, $e_{j}$ is the main effect of environment *j*, and $\varepsilon_{ijk}$ is residual error. In each simulation, the variance of the vector of additive genetic values within an environment was taken as environment‐specific genetic variance, $\sigma_{g(j)}^{2}$. Using the R function *rnorm*, values of $e_{j}$ were sampled from a normal distribution with a variance equal to twice $\sigma_{g(j)}^{2}$. Values of $\varepsilon_{ijk}$, were sampled from a normal distribution with a $\sigma =\sqrt{\sigma_{\varepsilon }^{2}}$, μ=0. Within each environment $\sigma_{\varepsilon }^{2}$ was determined based on the desired level of narrow‐sense heritability within the environment on a per‐plot basis $h_{j}^{2}$, for the simulation run. This variance was calculated as follows:

(3) $$\sigma_{\varepsilon }^{2}=\frac{1-h_{j}^{2}}{h_{j}^{2}}\;\sigma_{g\left(j\right)}^{2}$$

where $\sigma_{\varepsilon }^{2}$ is the within‐environment residual error variance, $h_{j}^{2}$ is a designated narrow‐sense heritability within $e_{j}$, $\sigma_{g(j)}^{2}$ is the genetic variance within $e_{j}$. Ultimately, at $e_{j}$ in a given simulation, the designated $h_{j}^{2}$ corresponds to:

(4) $$h_{j}^{2}=\frac{\sigma_{g\left(j\right)}^{2}}{\sigma_{g\left(j\right)}^{2}+\sigma_{\varepsilon }^{2}}$$

where variables carry the same definitions as in Equation (3).

Error variance ($\sigma_{\varepsilon }^{2}$) was calculated at the environment level to ensure both RR and CR faced equivalent residual error variance relative to genetic variance. To be conservative, area main effects, either block‐ or trial‐specific, were not simulated. Yet, area main effects can arise due to chance. Contrary to environment main effects (*e_j_*), area main effects would be cleanly estimable in CR designs but confounded with genetic effects in RR designs alongside $r_{k_{1}k_{2}}$ unless common checks are present between areas. To conservatively evaluate the merit of CR relative to RR, this simulation did not consider area main effects supposing that these effects could be estimated with the checks. Genotype‐specific performance varied between areas within a given environment solely through genotype‐by‐area interactions, implemented via correlated marker effects with correlation structure determined by $r_{k_{1}k_{2}}$. Beyond this genetic variation across areas, phenotypic variation among replicates of the same genotype within each area arose from plot‐level residual error ($\varepsilon_{ijk}$), which captured microenvironmental heterogeneity and measurement error. This approach enabled the evaluation of replication level, heritability ($h_{j}^{2}$), and $r_{k_{1}k_{2}}$ effects on selection accuracy (*r_bv_*) and avoided introducing assumptions that might favor CR over RR.

### 2.5 Scenarios simulated

Simulations were performed across a range of conditions that may be experienced in plant breeding trials. The controllable factors that we manipulated were the aspects of the testing design, which included the randomization scheme (CR or RR) and replication level. The uncontrollable factors that we manipulated were the $h_{j}^{2}$ and the genetic correlation between areas within environments ($r_{k_{1}k_{2}}$), creating genotype‐by‐area interaction (Table 2). We describe these factors as uncontrollable because they are largely beyond the control of researchers.

**TABLE 2:** Description of trials simulated at each location.

Randomization scheme	Design parameter set	Replication	Trial	Cohorts included	Trial design type	Blocks	Replicates per line within cohort			
							S1	S2	S3	S4
Restricted	1	High	Prelim	S1, S2	RCBD	2	2	2	0	0
			Adv	S3, S4	IBD	3	0	0	2	3
	2	Intermediate	Prelim	S1, S2	RCBD	2	2	2	0	0
			Adv	S3, S4	RCBD	2	0	0	2	2
	3	Low	Prelim	S1, S2	CRD	1	1	1	0	0
			Adv	S3, S4	RCBD	2	0	0	2	2
Complete	4	High	Multi‐cohort	S1, S2, S3, S4	IBD	3	2	2	2	3
	5	Intermediate	Multi‐cohort	S1, S2, S3, S4	RCBD	2	2	2	2	2
	6	Low	Multi‐cohort	S1, S2, S3, S4	*p‐*rep	2	1	1	2	2

Abbreviations: Adv, advanced; CRD, completely randomized design; IBD, incomplete block design; Prelim, preliminary; *p‐*rep, partially replicated design; RCBD, randomized complete block design; S1, stage one; S2, stage two; S3, stage three; S4, stage four.

For the controllable factors, we created six different design configurations, allowing us to evaluate three different levels of replication within each of the two randomization schemes. Within each of the randomization schemes, there were three different levels of replication, resulting in a total of six different within‐environment design configurations, where each replication level was comparable to its counterpart in either CR or RR (Figure 2). In design configurations one, two, and three that employed RR, there was a preliminary trial consisting of S1 and S2 cohorts and an advanced trial consisting of S3 and S4 cohorts (Table 1). Randomization was restricted within trial, which was also the designated area within environment in RR. Design configuration one contained high replication, where the S3 and S4 cohorts were replicated three times and the S1 and S2 were replicated twice, reflective of programs that may employ elite trials in addition to advanced and preliminary (Belamkar et al., 2018). Design configuration two was nearly the same as set one, except the replication was intermediate and balanced across cohorts, with all lines replicated twice. In design configuration three, the preliminary trial was not replicated, and the advanced trial was replicated twice.

In the design configurations four, five, and six that employed CR, there was only one trial within every set consisting of all four breeding cohorts, and resolvable blocks were designated as the area within environment. The level of replication in design configuration four was high, with S3 and S4 lines replicated three times and S1 and S2 lines replicated twice, comparable to RR set one. Design configuration five had intermediate replication with all lines replicated two times, comparable to RR set two. Design configuration six had low replication, with no replication of S1 and S2 lines and two replicates of S3 and S4 lines, comparable to RR set three.

Test plot numbers were held constant between corresponding RR and CR design configurations (Figure 2), with eight check plots allocated per block across all design configurations (four unique checks, replicated twice per block). The total entry numbers differed due to variations in check‐plot composition. The RR designs simulated more total checks per trial, a pattern that reflects the RR's reliance on design checks to support the blocking structure and the RR of cohorts within them. Conversely, CR's completely randomized structure inherently requires fewer blocks, and the simulated check numbers mirrored how this design obviates the need for design checks, with a reduced check line composition reflecting the use of performance checks alone.

For the uncontrollable factors, we simulated $h_{j}^{2}$ levels of 0.8, 0.6, 0.4, and 0.2. The levels of $r_{k_{1}k_{2}}$ simulations were 1.0, 0.8, 0.6, 0.4, and 0.2. When $r_{k_{1}k_{2}}=1$, there is no genotype‐by‐area interaction, and we expect no advantage of CR relative to RR. When $r_{k_{1}k_{2}}=0.2$ the genotype‐by‐area interaction is at its highest level, representing the most challenging scenario. All possible combinations of $h_{j}^{2}$, $r_{k_{1}k_{2}}$, and design configuration were considered, for a total of 120 different scenarios. For every scenario, we simulated five environments, assuming a genetic correlation between environments of 0.5. Conventional selection underwent 500 simulation runs for every unique scenario, while GS underwent 50 simulations due to computational load. In addition to these balanced multi‐environment testing scenarios, we mimicked two sparse‐testing multi‐environment trial designs, one with RR and one with CR. To do so, we used the low‐replication design configurations three and six but only allowed the S1 and S2 cohorts to be evaluated in one of the five environments. For every unique scenario considered in the sparse testing experiment, we performed 50 simulation runs.

### 2.6 Breeding value estimation

To estimate breeding values from each simulated dataset, we fit mixed models with environment‐specific genetic variances and environment‐specific block variances according to A. B. Smith et al. (2007):

(5) $$y_{ijk}=\mu +e_{j}+b_{k\left(j\right)}+g_{i\left(j\right)}+\varepsilon_{ijk}$$

where $y_{ijk}$ is the phenotypic value for the *i*th genotype in the *k*th block of the *j*th environment. The mean is μ, the fixed effect of environment *j* is $e_{j}$, the random effect of block *k* nested within environment *j* is $b_{k(j)}$ with $b_{k(j)}∼N(0,\;B$), where **B** contains environment‐specific block variances on the diagonal. The random effect of genotype *i* within environment *j* is $g_{i(j)}$. We assumed $g_{i(j)}∼N(0,\;G_{g}$). The variance structure $G_{g}$ was given by:

(6) $$G_{g}=G_{e}\;⊗\;G_{m}$$

where $G_{g}$ is an *i* × *i* genetic variance matrix with environment‐specific genetic variances on the diagonals. The covariance matrix $G_{m}$ is a m×m genetic covariance matrix for lines. We had two assumptions for $G_{m}$. First, lines were assumed unrelated and $G_{m}$ was an identity matrix. We refer to this method as “conventional selection.” Next, lines were assumed to be related and $G_{m}$ was a GRM calculated previously. We refer to this method as GS. Models were implemented in ASREML‐R Version 4.2 (Butler et al., 2018).

The conventional selection model was evaluated for all 120 scenarios, as the absence of marker data permitted this comprehensive assessment with reasonable computational load. For GS, we examined the low replication scenarios, including sparse testing, because these are of greatest interest to plant breeders performing GS and because interactions with the randomization scheme and replication level were not detected in our initial analyses using a conventional selection model. In total, GS accuracies were evaluated for 40 unique simulation scenarios across the varying levels of $h_{j}^{2}$ and $r_{k_{1}k_{2}}$. In each simulation and for each analysis method, selection accuracy was assessed using a Pearson's correlation between the breeding value estimates and the true breeding values simulated, referred to as *r_bv_*. For conventional selection and GS, *r_bv_* was evaluated across all cohorts within a simulation. For genomic‐enabled sparse testing, *r_bv_* was evaluated for only the test set, containing only the untested S1 and S2 cohorts. $\bar{r}$_bv_ was calculated by averaging across all *r_bv_* calculated from all individual runs of a simulation.

### 2.7 Analyses of simulation results

Analysis of variance (ANOVA) was performed to examine the main effects of randomization scheme, $h_{j}^{2}$, replication level, and $r_{k_{1}k_{2}}$ on selection accuracy. The ANOVA model included all main effects plus two‐way interactions between randomization scheme and each of the other factors, as described by the following equation:

(7) $$\begin{array}{ccc} ry_{\mathrm{ijklm}} & = & \mu +z_{i}+h_{j}+r_{k}+\rho_{l}+{\left(h\times z\right)}_{\mathrm{ik}}+{\left(r\times z\right)}_{\mathrm{jk}}\\ & & +\;{\left(\rho \times z\right)}_{\mathrm{kl}}+\nu_{m}+\varepsilon_{\mathrm{ijklm}} \end{array}$$

where $y_{ijklm}$ is the observed selection accuracy, μ is the overall mean; $z_{i}$, $h_{j}$ $r_{k}$, $\rho_{l}$ are the fixed main effects of the *i*th randomization scheme of either CR or RR, *j*th heritability level, the *k*th $r_{k_{1}k_{2}}$ level and *l*th replication level; ${\left(h\times z\right)}_{ik}$, ${\left(r\times z\right)}_{jk}$, ${\left(\rho \times z\right)}_{kl}$, are the fixed two way interaction effects between randomization scheme z and each of heritability, $r_{k_{1}k_{2}}$, and replication level, respectively, $\nu_{m}∼N\left(0,\sigma_{\nu }^{2}\right)$ is the random effect of the *m*th simulation run accounting for stochastic variability across independent runs, and *ϵ_ijklm_* is the residual error term assumed to be independent and identically distributed. For GS and sparse testing analyses, where only low replication scenarios were evaluated, the ANOVA was updated to omit the replication factor $\rho_{l}$ from both the main and interaction effect terms.

To quantify how selection accuracies differ for RR and CR across a range of factors, we employed difference‐in‐differences (DiD) estimation, allowing us to isolate randomization scheme‐specific responses as experimental conditions deteriorate from optimal. This approach requires three key assumptions that our simulation satisfies: (1) consistency through well‐defined treatment groups, (2) positivity through equal allocation of genotypes and factor levels across designs, and (3) parallel trends demonstrated by equivalent performance (±0.001 $\bar{r}$ *_bv_*) under optimal conditions (Rothbard et al., 2024). Establishing these parallel trends requires designating control and intervention groups that perform equivalently at baseline; subsequent divergence can then be causally attributed to differential responses rather than pre‐existing differences. Following this DiD convention, we assigned the established practice (RR) as the control group and the alternative approach (CR) as the intervention group. The optimal factor combination that serves as our baseline of equivalent performance was $h_{j}^{2}$ = 0.8, high replication, and $r_{k_{1}k_{2}}$= 1, representing the best‐case scenario for a breeding program in which selection accuracies are highest. In this case, parallel trends are achieved given that selection accuracies are equivalent for both RR and CR at baseline. Post‐intervention conditions were those with lower levels of $h_{j}^{2}$, replication, and $r_{k_{1}k_{2}}$. The DiD model was specified as follows:

(8) $$E\;\left(Y|Z,\;T\right)=\beta_{0,t}+\beta_{1}I\left(T=1\right)+\beta_{2}Z+\beta_{3}I\left(T=1\right)Z$$

where the expectation operator, E(Y|Z,T), is the expected selection accuracy *Y*, for any individual simulation run given the randomization scheme where *Z*_0_ = control group, RR, and *Z*_1_ = intervention group, CR, and factor conditions where *T* = 0 for optimal, that is, pre‐treatment baseline, and *T* = 1 for suboptimal, that is, post‐treatment. $\beta_{0,t}$ is the baseline intercept for optimal conditions of our control group RR, $\beta_{1}I\left(T=1\right)$ is the effect of suboptimal conditions on the prediction accuracies in the control group RR, $\beta_{2}Z$ is the expected difference between prediction accuracies of control RR and intervention CR under optimal conditions, and finally, our coefficient of interest *δ̂*_DiD_, the DiD estimator, is represented by *β*_3_, and quantifies the differential response between randomization schemes as a given factor ($h_{j}^{2}$, $r_{k_{1}k_{2}}$, or replication level) progressively deteriorates from optimal to suboptimal. Positive DiD values indicate greater performance of the intervention group, CR, relative to the control group, RR, as experimental conditions become less conducive to higher prediction accuracies.

## 3 RESULTS AND DISCUSSION

### 3.1 Population structure among the breeding lines was slight

**FIGURE 3:**
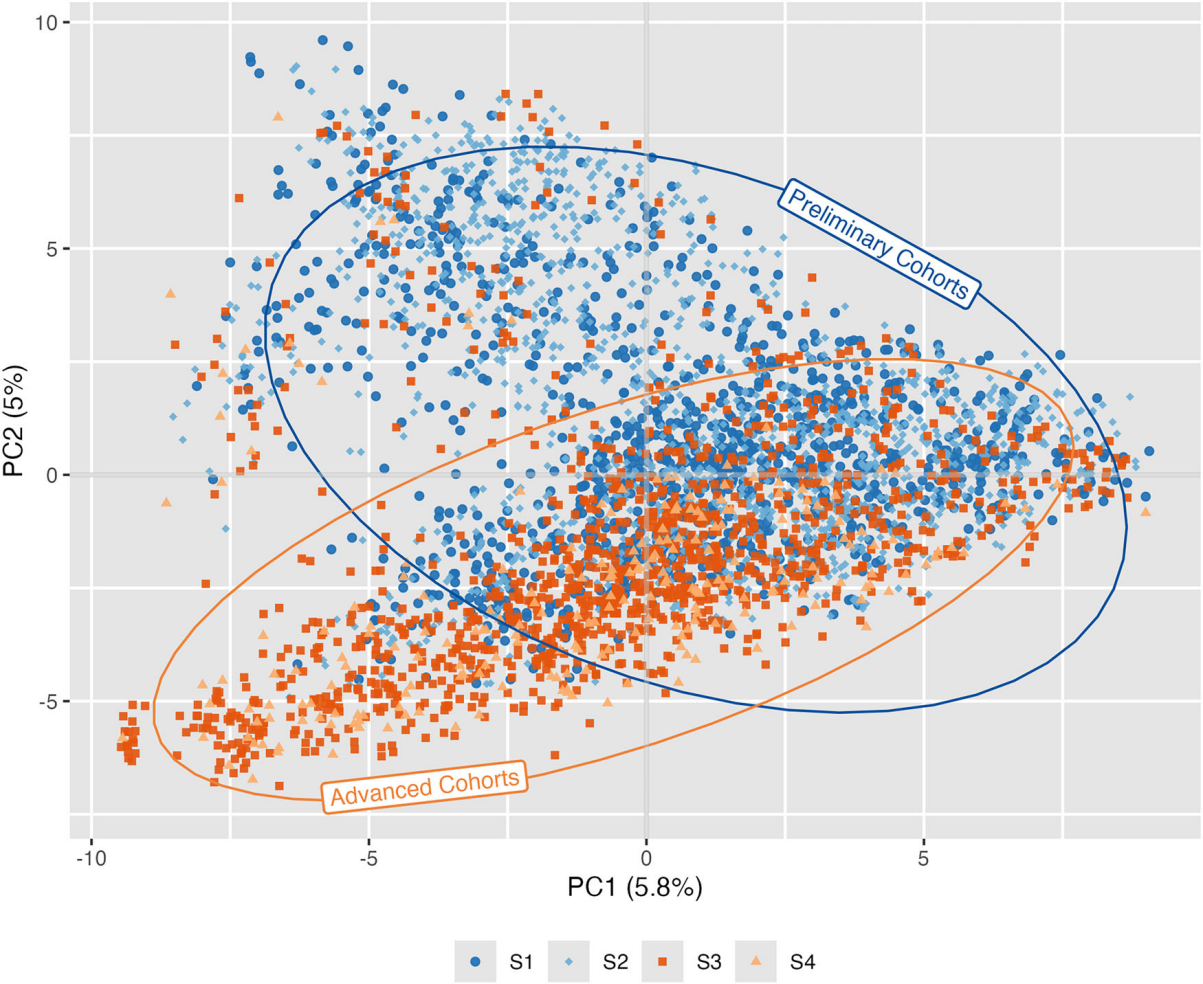
Principal component analysis based on the genomic relationship matrix. Principal component one (PC1) and principal component two (PC2) computed from the additive genomic relationship matrix of the 4102 wheat lines used in this study are plotted on the *x* and *y*‐axis, respectively. The first two principal components explain 5.8% and 5% of the variation. Lines are color‐coded indicating to which cohort they belong. Cohort S1, S3, S3, and S4 are indicated with dark blue circles, light blue diamonds, red squares, and yellow triangles, respectively. Lines clustering with the preliminary cohorts (S1 and S2) are circled in blue, and lines clustering with the advanced cohorts (S3 and S4) are circled in orange.

Little to no population structure was observed among our breeding lines. The PCA revealed that PC1 and PC2 accounted for only 5.8% and 5% of the total variation, respectively (Figure 3). These relatively low percentages are not surprising because all lines belong to four consecutive cohorts of a single breeding program. Nevertheless, some separation of preliminary and advanced breeding lines is evident, which is expected given that lines of different cohorts were developed from different, albeit related, sets of parental lines. The genetic differences between preliminary and advanced lines are expected to lead to confounding between genetic and trial effects when these groups are evaluated in separate trials.

### 3.2 Conventional selection accuracy improved with CR

**FIGURE 4:**
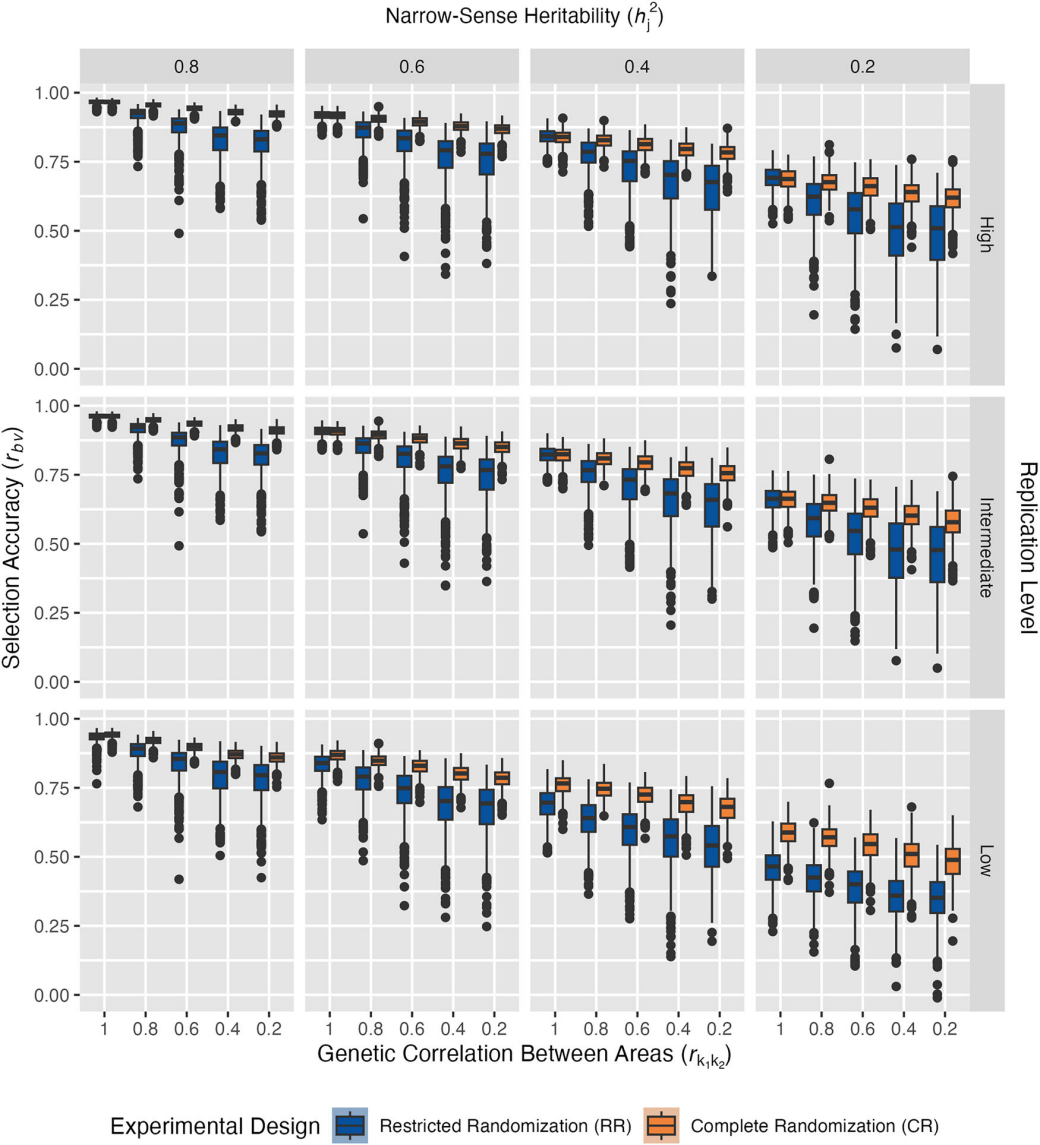
Conventional selection accuracy across experimental factors. Box‐and‐whisker plots show distributions of best linear unbiased prediction (BLUP) accuracies across 500 simulations in restricted randomization (RR, blue) or complete randomization (CR, orange) as *r_bv_* decreases along the *y*‐axis. Panels are arranged by narrow‐sense heritability within environment $h_{j}^{2}$, which ranged from 0.8 to 0.2, and replication level, which was either low, intermediate, or high. Genetic correlation between areas, $r_{k_{1}k_{2}}$, is shown decreasing across the *x*‐axis, representing increasing genotype‐by‐area interaction.

**FIGURE 5:**
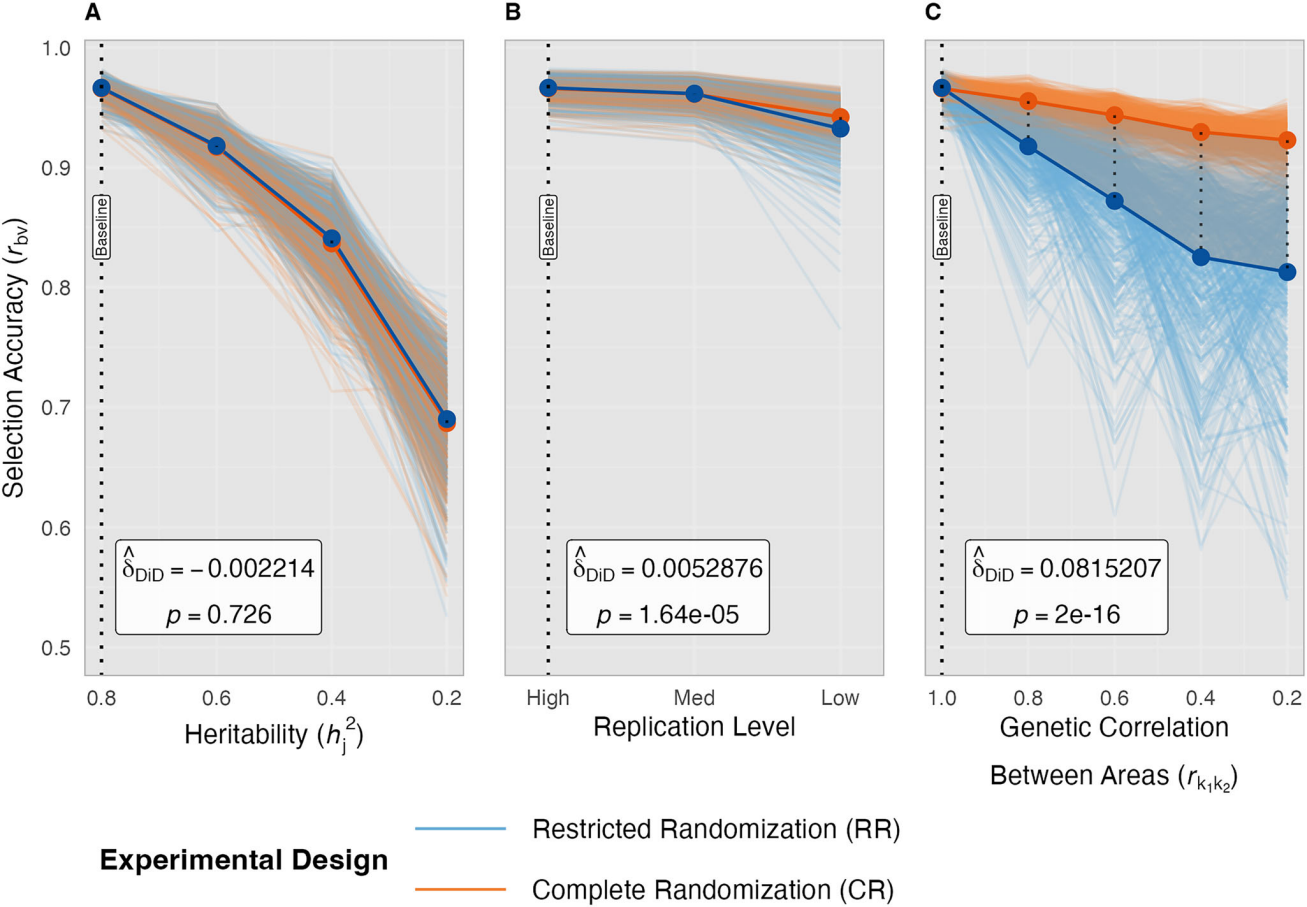
Difference‐in‐differences (DiD) analysis of conventional selection accuracies. Accuracy of best linear unbiased prediction (BLUP), *r_by,_* is shown along the *y*‐axis for restricted randomization (RR, blue; control) and complete randomization (CR, orange; intervention) as experimental conditions progressively deteriorate from an optimal baseline, moving from left to right on the *x*‐axes. Narrow‐sense heritability within environment, $h_{j}^{2}$, panel A; Deterioration in replication level, panel B, and genetic correlation between areas $r_{k_{1}k_{2}}$, panel C is examined individually while keeping the other parameters at their optimal levels. Light lines denote individual simulation runs (*n* = 500 per scenario), while bold lines show the overall mean trend of the given design. The vertical dotted line (“baseline”) marks the optimal factor combination ($h_{j}^{2}$ = 0.8, high replication, $r_{k_{1}k_{2}}$ = 1), at which RR and CR perform equivalently (*r_bv_* ≈ 0.97), supporting the parallel‐trends condition for causal interpretation. Shaded regions between design lines illustrate the magnitude of difference in *r_bv_*, with changes in the width of these regions relative to baseline visualizing the DiD effect.

Conventional selection accuracies were higher for CR than RR (Figure 4), but their relative performance depended on the level of $r_{k_{1}k_{2}}$, and to some extent $h_{j}^{2}$ and replication level (Figure 5). ANOVA confirmed that all main and interaction effects of factors in our simulation, including the randomization scheme, had highly significant main effects on conventional selection accuracy (p ≤ 0.001, Table 3). Among the main effects, $h_{j}^{2}$ exerted the largest influence on selection accuracy, followed by randomization scheme and replication level, while $r_{k_{1}k_{2}}$ showed the smallest main effect. However, the interaction effects revealed a contrasting pattern. Among the interactions with randomization scheme, $r_{k_{1}k_{2}}$ exhibited the strongest interaction effect on selection accuracy, followed by replication level, while the $h_{j}^{2}$ × randomization scheme interaction was comparatively weak (Table 3). This pattern suggests that $r_{k_{1}k_{2}}$ is a key determinant of whether randomization scheme influences selection outcomes, despite having a smaller main effect on selection accuracy than $h_{j}^{2}$, replication level, and randomization scheme alone.

**TABLE 3:** Multi‐factor analysis of variance (ANOVA) tables examining the factors affecting selection accuracy in our simulations with all lines evaluated in all six environments.

Selection method	Source	Degrees of freedom	Mean squares	*F*‐value	Significance
Conventional selection	$h_{j}^{2}$	3	335.0	83,902.1	<2e‐16
	$r_{k_{1}k_{2}}$	4	28.2	7053.7	<2e‐16
	Randomization scheme	1	100.8	25,237.8	<2e‐16
	Replication level	2	49.2	12,323.8	<2e‐16
	$h_{j}^{2}$ × Randomization scheme	2	1.4	356.9	<2e‐16
	$r_{k_{1}k_{2}}$ × Randomization scheme	3	5.0	1258.9	<2e‐16
	Replication level × randomization scheme	2	1.3	320.2	<2e‐16
	Error	59,979	0.004	–	–
Genomic selection	$h_{j}^{2}$	3	1.0664	2292.26	< 2e‐16
	$r_{k_{1}k_{2}}$	4	0.4182	898.963	< 2e‐16
	Randomization scheme	1	0.0048	10.333	0.00133
	$h_{j}^{2}$ × Randomization scheme	3	0.003	0.687	0.55995
	$r_{k_{1}k_{2}}$ × Randomization scheme	4	0.0010	2.231	0.06339
	Error	1983	0.0005	–	–
Genomic selection with sparse testing	$h_{j}^{2}$	3	2.1638	826.093	< 2e‐16
	$r_{k_{1}k_{2}}$	4	0.4391	167.635	< 2e‐16
	Randomization scheme	1	0.1108	42.307	9.11e‐11
	$h_{j}^{2}$ × Randomization scheme	3	0.0021	0.801	0.493
	$r_{k_{1}k_{2}}$ × Randomization scheme	4	0.0186	7.087	1.12e‐05
	Error	2983	0.0026	–	–

*Note*: $h_{j}^{2}$ is the narrow sense heritability within environment, $r_{k_{1}k_{2}}$ is the genetic correlation between areas.

Under optimal conditions ($h_{j}^{2}$ = 0.8, high replication, and $r_{k_{1}k_{2}}$= 1), both RR and CR achieved identical selection accuracies ($\bar{r}$ *_bv _* = 0.97, Figure 4). While such ideal conditions are unachievable, this idealized baseline enables us to make causal inferences about how randomization schemes respond differently to deteriorating experimental conditions (Table 4). The observed equivalent baseline performance satisfies the parallel trends assumption necessary for DiD, allowing subsequent divergence between CR and RR to be directly attributed to their differential responses to specific factor manipulations rather than pre‐existing performance gaps.

**TABLE 4:** Difference‐in‐differences (DiD) coefficients and their corresponding *p*‐values showing which simulation parameters significantly impacted the difference in performance between randomization schemes for a given selection method.

Selection method	Source	DiD coefficient	*p*‐value
Conventional selection	$h_{j}^{2}$	−0.002	0.726
	Replication level	0.005	1.64e‐05
	$r_{k_{1}k_{2}}$	0.082	<2e‐16
Genomic selection	$r_{k_{1}k_{2}}$	0.002	0.653
Genomic selection with sparse testing	$r_{k_{1}k_{2}}$	0.018	0.07

*Note*: $h_{j}^{2}$ is the narrow sense heritability within environment, $r_{k_{1}k_{2}}$ is the genetic correlation between areas.

Above all parameters, $h_{j}^{2}$ was shown to exert the strongest main effect on selection accuracy; however, its influence remained largely consistent across randomization schemes (small $h_{j}^{2}$ × randomization scheme interaction effect, Table 3). This independence is reflected by the nearly identical response slopes between RR and CR, differing by only 0.01% on average for each level of $h_{j}^{2}$ tested (Figure 4). The DiD analysis further confirms this independence (Figure 5A), yielding non‐significant interaction coefficients (*δ̂*_DiD_, *p* ≥ 0.10) between $h_{j}^{2}$ and randomization scheme, indicating no differential response between CR and RR as $h_{j}^{2}$ decreased. However, it is important to keep in mind that in practice, it is likely that low $r_{k_{1}k_{2}}$ would be observed together with low $h_{j}^{2}$. In this DiD analysis, we fixed $r_{k_{1}k_{2}}$ = 1.0 to examine the effects of $h_{j}^{2}$ independent of $r_{k_{1}k_{2}}$.

For replication levels, both CR and RR were equally sensitive to reduction from high to intermediate levels. However, when replication decreased further to low levels, selection accuracies suffered more with RR than CR, declining −2.98% with RR but only −1.94% with CR (Figure 4). Low replication contains unreplicated entries, distinct from high and intermediate replication levels, where entries are replicated at least twice (Figure 2; Table 2). These results agree with research demonstrating that benefits from replication follow a logarithmic relationship, where the first replication provides substantially greater gains in statistical power than subsequent ones (Yan, 2021). ANOVA showed an extremely significant interaction between replication level and randomization scheme (*p* ≤ 0.001, Table 3). The DiD analysis further confirmed that the differential response between selection accuracies with RR and CR that arose as replication levels decreased was extremely significant (*p* ≤ 0.001, Figure 5B). However, the DiD coefficient was negligible, only corresponding to an estimated 0.5% increase in accuracy for CR as the replication level decreased (*δ̂*_DiD_ = 0.005, Figure 5B). This pattern aligns with the logarithmic relationship between replication and selection accuracy, where both randomization schemes responded similarly to initial replication reduction (high to intermediate) but diverged meaningfully only when replication dropped to unreplicated levels (low). These results are expected because low replication reduces the precision of variance component estimates, particularly when unreplicated entries are included, making accurate partitioning of genetic and error variances more difficult.

As $r_{k_{1}k_{2}}$ decreased from 1.0 to 0.2, CR became increasingly advantageous, transforming from equal performance at $r_{k_{1}k_{2}}=1$ to substantial CR advantages of +8.8%, +18.0%, and +18.6%, corresponding to $r_{k_{1}k_{2}}$ = 0.6, 0.4, and 0.2, respectively (Figure 4). This finding is strongly supported by the DiD analysis results (Figure 5C), which confirmed that the differential response in selection accuracy between randomization schemes was due to the progressive deterioration of $r_{k_{1}k_{2}}$ (*p* ≤ 0.001). The DiD coefficient was substantial (*δ̂*_DiD_ = 0.082), corresponding to an estimated 8.2 percentage point advantage for CR over RR with each unit decrease in $r_{k_{1}k_{2}}$. These results strongly suggest that when different cohorts are to be analyzed together for selection in the absence of genomic relationship information, they should be randomized together.

### 3.3 GS accuracy was unaffected by randomization scheme under complete testing

**FIGURE 6:**
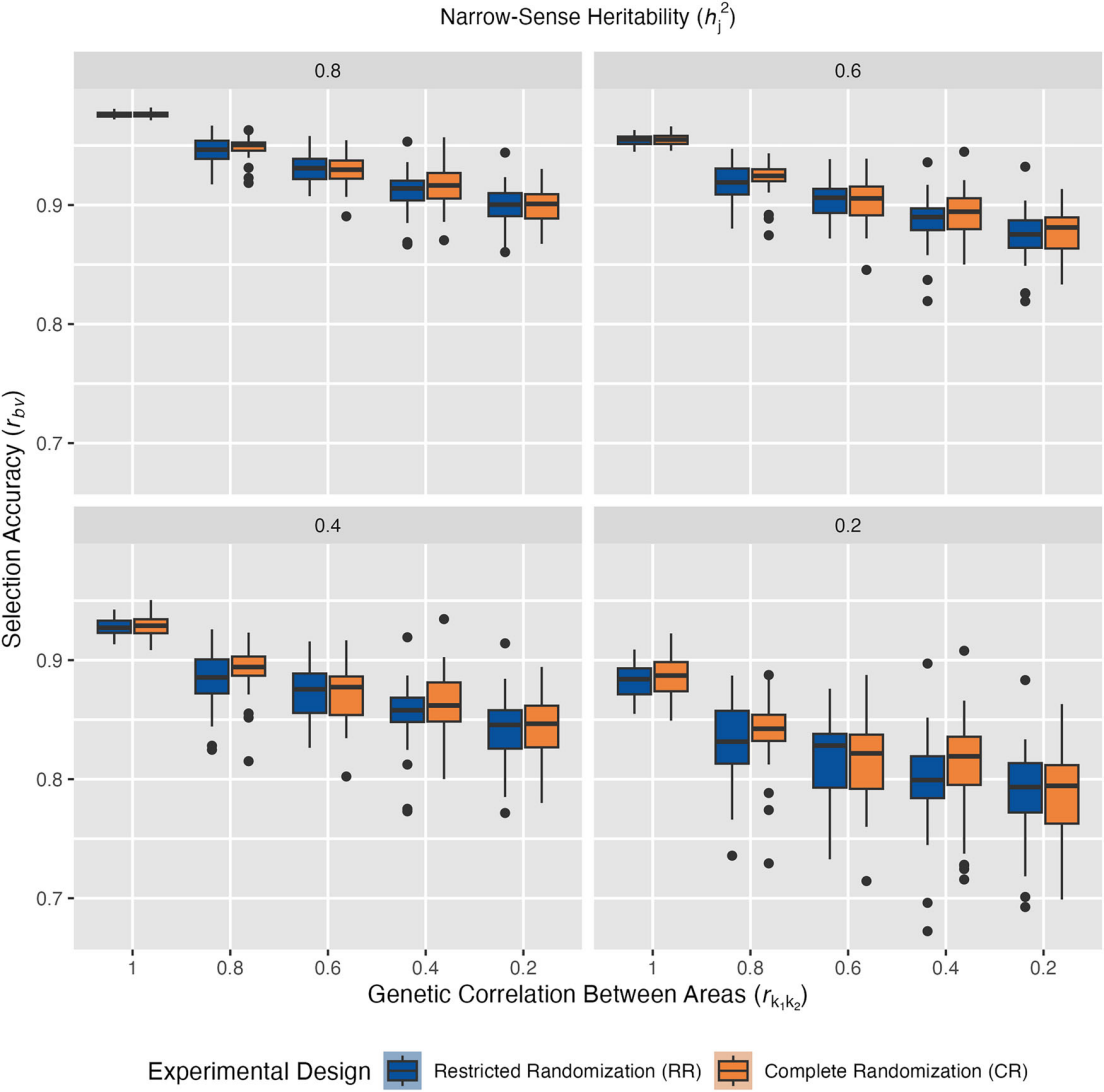
Genomic selection accuracy under low replication across experimental factors. Box‐and‐whisker plots show the distributions of genomic best linear unbiased prediction (GBLUP) accuracies across 50 simulations in restricted randomization (RR, blue) or complete randomization (CR, orange) with *r_bv_* along the *y*‐axis. Panels are arranged by narrow‐sense heritability within environment, $h_{j}^{2}$, ranging from 0.8 to 0.2. Genetic correlation between areas, $r_{k_{1}k_{2}}$, is shown decreasing across the *x*‐axis, representing increasing genotype‐by‐area interaction.

When all cohorts were evaluated across all five environments and genomic relationship information was incorporated through GBLUP, the confounding between genetic and non‐genetic effects due to RR was effectively mitigated. The GRM provided connectivity between spatially separated cohorts that reduced design‐dependent effects on GS accuracy. Both CR and RR achieved nearly identical GS accuracies across all levels of $h_{j}^{2}$ and $r_{k_{1}k_{2}}$ examined (Figure 6). Mean GS accuracies differed by <0.5% between designs across all scenarios, with both approaches maintaining high accuracy even under challenging conditions of low heritability and strong genotype‐by‐area interaction.

ANOVA confirmed that while randomization scheme significantly influenced GS accuracy (*p* ≤ 0.001, Table 3), its impact was substantially smaller compared to that of conventional selection. Main effects for experimental factors were significant (*p* ≤ 0.001), and like with conventional selection, the main effect for $h_{j}^{2}$ exerted the largest influence on GS accuracy. However, the ranking of the remaining factors reversed: $r_{k_{1}k_{2}}$ demonstrated greater influence on GS accuracy than the randomization scheme, whereas the randomization scheme exceeded $r_{k_{1}k_{2}}$ for conventional selection. This pattern reflects not a heightened impact of $r_{k_{1}k_{2}}$ in GS, but rather a negligible effect of randomization scheme in this context. A key finding was the absence of significant interactions between $r_{k_{1}k_{2}}$ and randomization scheme, as well as between $h_{j}^{2}$ and randomization scheme (*p *> 0.10). Furthermore, effect sizes for both main and interaction effects involving $h_{j}^{2}$, $r_{k_{1}k_{2}}$, and randomization scheme were substantially smaller with GS than with conventional selection (Table 3). These results indicate that the inclusion of genomic information effectively mitigated the harm of RR on selection accuracy found in conventional selection.

**FIGURE 7:**
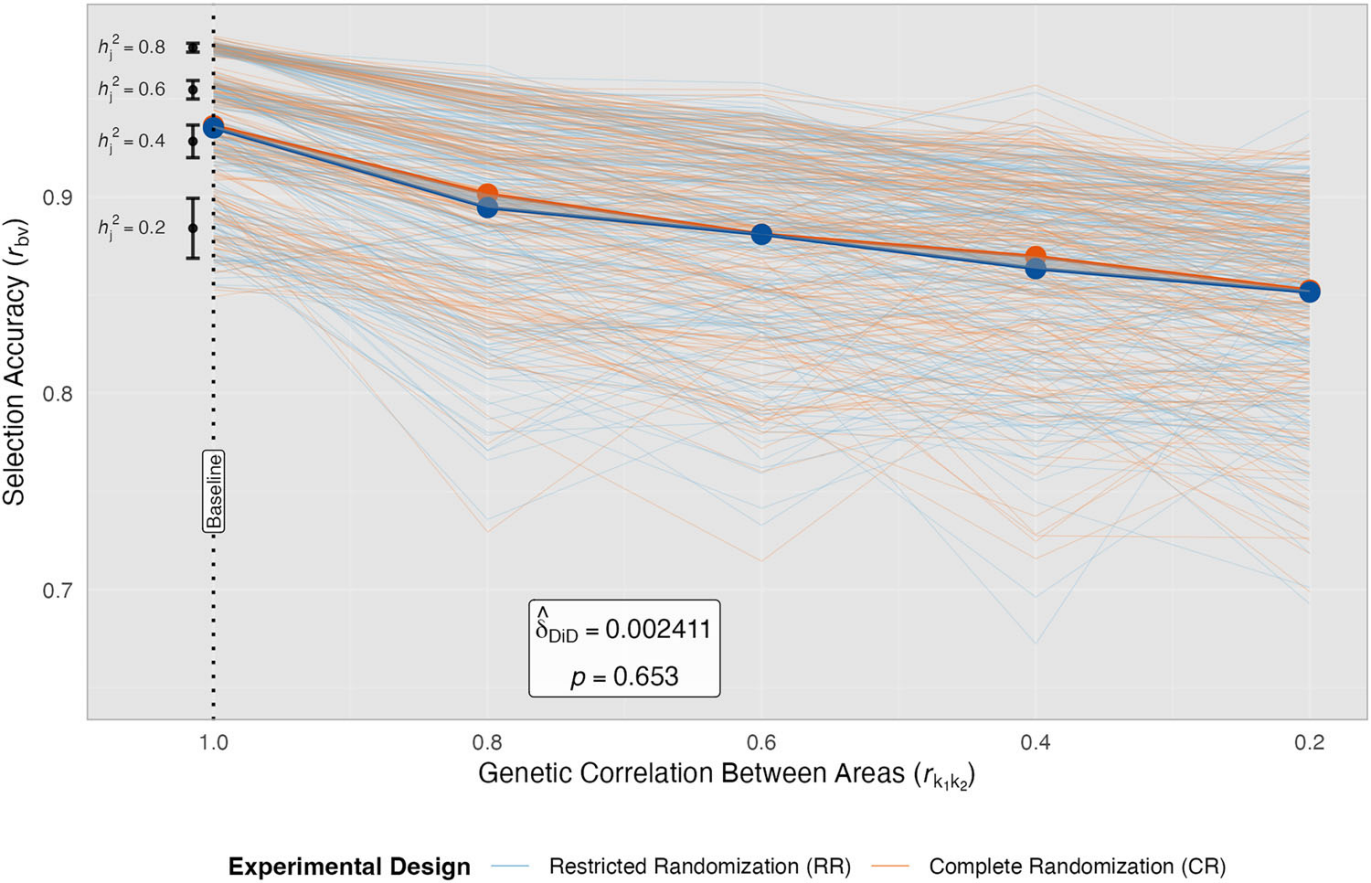
Difference‐in‐differences analysis of genomic selection under low replication. Accuracy of genomic best linear unbiased prediction (GBLUP), or *r_by_*, is shown along the *y*‐axis for restricted randomization (RR, orange) and complete randomization (CR, blue) designs. Light lines represent individual simulation runs (*n *= 50 per scenario), while bold lines show the overall mean trends of either design. Black error bars at the left side of the plot display heritability‐specific means ± standard deviation, with corresponding within environment narrow sense heritability, $h_{j}^{2}$, values labeled on the left. The vertical dotted line marks the optimal baseline conditions where both designs achieve near‐equivalent performance, supporting parallel trends assumptions for causal inference. Shaded regions between design trendlines illustrate the magnitude of difference in *r_bv_* as the genetic correlation between areas, $r_{k_{1}k_{2}}$, progressively deteriorates along the *x*‐axis past the intervention point, with minimal change in the width of these regions from baseline, illustrating the non‐significant difference‐in‐differences (DiD) effect.

The DiD analysis results further confirmed the absence of meaningful differences between randomization schemes for GS with complete testing (Figure 7; Table 4). As $r_{k_{1}k_{2}}$ progressively deteriorated, the differential response in GS accuracy between CR and RR was found to be non‐significant (*p *> 0.10), indicating that CR provided no measurable advantage over RR even as genotype‐by‐area interaction increased. This stark contrast with conventional selection (Figure 5C) demonstrates that the GRM can effectively reduce the confounding between genetic and environmental effects that occurs under RR when cohorts are restricted to separate trials. These findings suggest that breeding programs routinely incorporating GRMs in their analyses with balanced multi‐environment testing can use either CR or RR without compromising selection accuracy. The ability of the GRM to capture the genetic covariance structure among individuals across cohorts eliminates the need for CR as a mechanism to prevent genetic and non‐genetic confounding, at least when all genotypes are evaluated in all environments. However, it is important to point out that how well the GRM can reduce confounding in RR testing schemes depends on the extent to which the genotypes evaluated are genetically related.

### 3.4 CR improved GS accuracy under sparse testing

**FIGURE 8:**
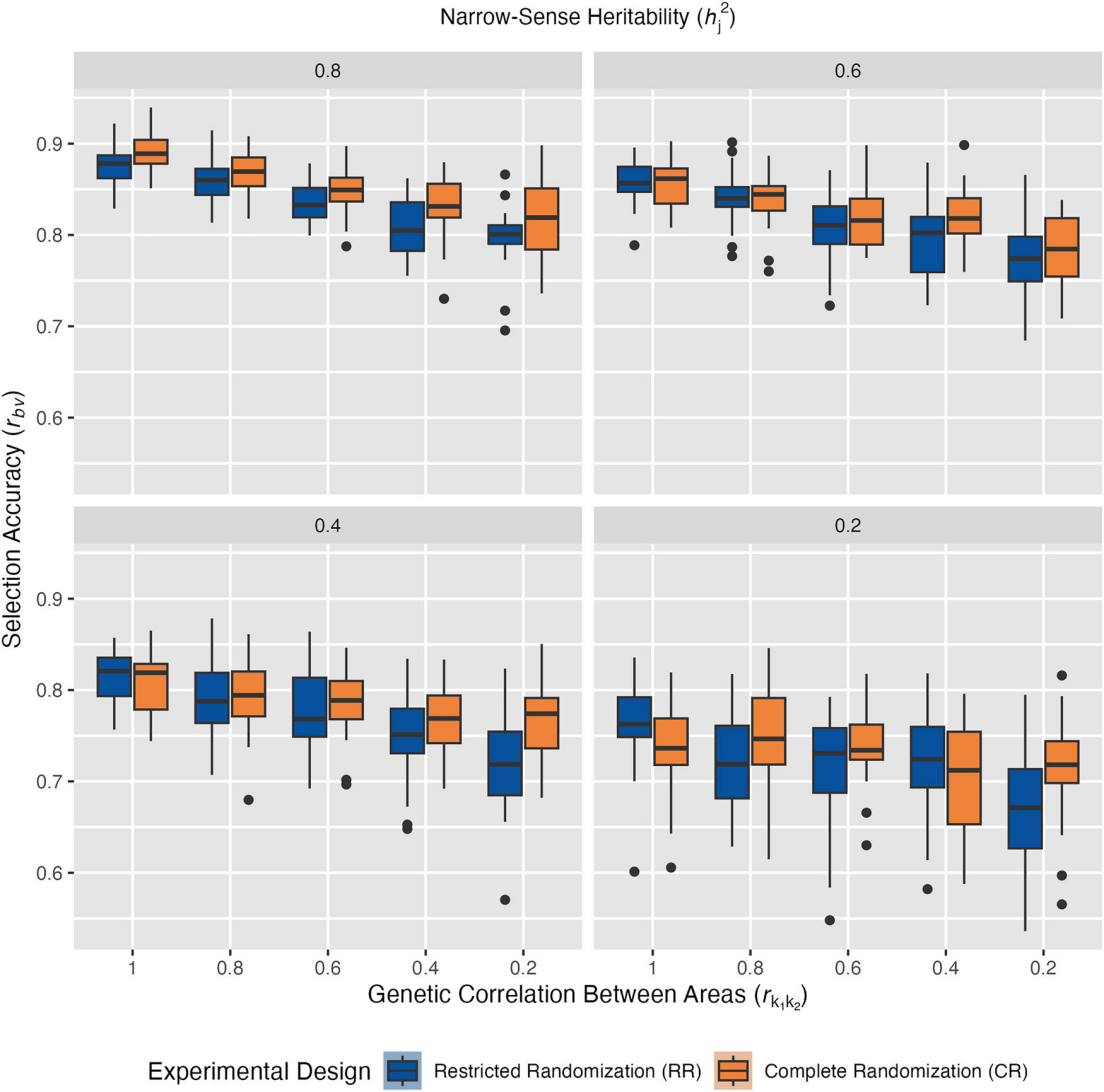
Genomic selection accuracy of sparse testing designs under low replication across experimental factors. Box‐and‐whisker plots show distribution of genomic best linear unbiased prediction (GBLUP) accuracies, *r_bv_*, across 50 simulations in restricted randomization (RR, blue) or complete randomization (CR, orange) with *r_bv_* along the *y*‐axis, where, for a given run: S3 and S4 cohorts are evaluated in all environments, S1 and S2 cohorts are evaluated in only one of five environments, and *r_bv_* is calculated based on the predictive ability of GBLUP for S1 and S2 cohorts in the four untested environments. Panels are arranged by narrow‐sense heritability within environment, $h_{j}^{2}$, which ranged from 0.8 to 0.2. Genetic correlation between areas, $r_{k_{1}k_{2}}$, decreases across the *x*‐axis, representing increasing genotype‐by‐area interaction.

In stark contrast to complete testing where genomic information nearly eliminated design dependency, sparse testing revealed differences in design performance, with GS accuracies lower under RR than CR. In CR, GS accuracies exceeded those of RR by 1.5% across all simulated scenarios (Figure 8). At times, GS accuracies with RR were slightly higher than CR when $r_{k_{1}k_{2}}=1$, but the comparative advantage of CR increased as $r_{k_{1}k_{2}}$ decreased. Under the most challenging scenario of low $h_{j}^{2}=0.2$ and $r_{k_{1}k_{2}}=0.2$, GS accuracies from CR were 5.5% higher than those of RR.

In sparse testing scenarios, $h_{j}^{2}$ remained the dominant main effect on selection accuracy (*p* ≤ 0.001, Table 3), consistent with results from conventional selection and GS with complete testing. The interaction between $h_{j}^{2}$ and randomization scheme, however, remained non‐significant (*p* ≥ 0.10, Table 3), maintaining the pattern observed in GS with complete testing. However, a key finding emerged: the $r_{k_{1}k_{2}}$ × randomization scheme interaction returned to statistical significance under sparse testing (*p* ≤ 0.001, Table 3). Both the magnitude of this interaction effect and the main effect of randomization scheme positioned sparse testing as an intermediate case, exhibiting greater robustness to randomization scheme than conventional selection, yet more sensitivity to randomization scheme than GS with complete testing. These results indicate that $r_{k_{1}k_{2}}$ resumes its role as a key determinant of whether randomization scheme influences GS accuracy when phenotypic connectivity across environments is reduced through sparse testing.

**FIGURE 9:**
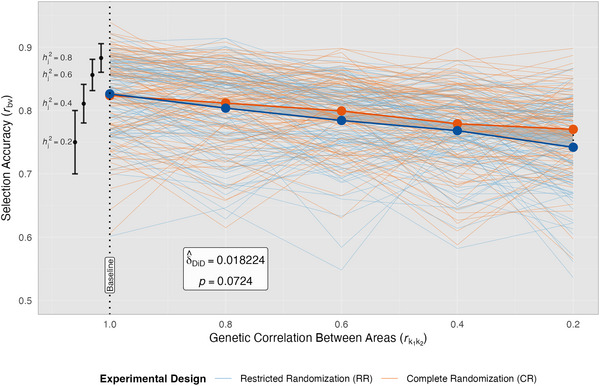
Difference‐in‐differences analysis of genomic selection accuracy for sparse testing designs under low replication. Accuracy of genomic best linear unbiased prediction (GBLUP), *r_by_*, is shown along the *y*‐axis for restricted randomization (RR, orange) and complete randomization (CR, blue); where, for a given run: S3 and S4 cohorts are evaluated in all environments; S1 and S2 cohorts are evaluated in only one of five environments, and *r_bv_* is calculated based on the selection accuracy of GBLUP for S1 and S2 cohorts in the four untested environments. Light lines represent individual simulation runs (*n* = 50 per scenario) for CR and RR designs, while bold lines show overall mean trends. Black error bars at the left side of the plot display heritability‐specific means ± standard deviation, with corresponding within‐environment heritability $,\;h_{j}^{2}$, values labeled on the left. The vertical dotted line marks the optimal baseline conditions where both designs achieve near‐equivalent performance, supporting parallel trends assumptions for causal inference. Shaded regions between design trendlines illustrate the magnitude of difference in *r_bv_* as genetic correlation between areas, $r_{k_{1}k_{2}}$, progressively deteriorates along the *x*‐axis past the intervention point, with the widening of this region from baseline visualizing the significant difference‐in‐differences (DiD) effect.

The DiD analysis results further supported this finding, showing that as $r_{k_{1}k_{2}}$ progressively deteriorated, the differential response in selection accuracy between CR and RR became marginally significant (0.05 < *p* ≤ 0.10, Figure 9). The modest DiD coefficient (*δ̂*_DiD _= 0.018) corresponded to CR maintaining an estimated 1.8 percentage points higher GS accuracy than RR for each unit decrease in $r_{k_{1}k_{2}}$. These DiD results contrast with those observed for GS with a balanced multi‐environment testing design, where designs performed almost identically with non‐significant DiD coefficients when $r_{k_{1}k_{2}}$ was the examined factor.

### 3.5 GS accuracy only sometimes benefited from CR

Altogether, these findings suggest that the addition of a GRM can nearly eliminate the genetic and non‐genetic confounding due to RR in complete multi‐environment testing designs, but when lines are not tested in all environments and RR is utilized within environments, the confounding effect becomes too great for the GRM to fully eliminate. The advantage of CR when GS is performed with sparse testing fell between that of conventional selection and GS with complete testing across all environments. This pattern reflects reduced connectivity between environments under sparse testing, which increases confounding between genetic and non‐genetic factors, ultimately impacting GS accuracies. Recall that sparse testing in this simulation implied that lines belonging to the S1 and S2 cohorts were only tested in one of the five environments, aligning these results with that of Atanda et al. (2022), who demonstrated that experimental design directly affects how effectively genomic prediction models leverage relationship matrices to predict performance in untested locations under sparse testing. Completely randomized designs mitigated these challenges by improving variance component estimation despite G×E interaction and unbalanced testing across locations, demonstrating that CR offers tangible benefits in the genomic‐enabled sparse testing designs that are becoming increasingly common in breeding programs.

## 4 CONCLUSION

Our simulation study demonstrates that randomization schemes within experimental designs remain consequential even in the GS era, with impact varying based on data completeness and the extent to which genotype performance varies across areas and environments. Two major conclusions from this study emerged. First, the advantage gained from CR depended on connectivity between areas within and across environments. When data connectivity is high, either randomization scheme performs well. However, when data are less connected, either through sparse testing or absence of genomic relationship information, CR outperforms RR, and this performance disparity grows as experimental conditions become less forgiving, showing up to 15.7% higher conventional selection accuracy in simulations with low replication (Figure 4). Second, $r_{k_{1}k_{2}}$ is the primary driver of performance differences between RR and CR (Figure 5). In situations where higher genotype‐by‐area interaction is present, such as when different areas are sown on different dates, experience different management, or have different soil types, CR designs could be especially advantageous.

These findings translate to practical recommendations for breeding programs that wish to obtain accurate breeding value estimates from analyses across multiple breeding cohorts, including from GS models: (1) for programs that routinely use GRMs in analyses and work with mostly balanced multi‐environment testing data, either randomization scheme can be used to obtain accurate predictions (Figures 6 and 7); (2) for programs that do not genotype routinely (Figures 4 and 5) or those that are aiming to optimize their resource allocation using sparse testing (Figures 8 and 9), CR is recommended. Although this research demonstrates that restrictions on randomization within experimental designs in a breeding program can impact a breeder's accuracy of selection across multiple cohorts, there are additional factors to consider when deciding to switch from RR to CR. Some breeders prefer RR to facilitate visual comparisons between individuals within the same cohort or to more easily take specific data or grain samples on a specific cohort of interest. Although CR does not prevent visual comparisons or selective data collection, it can substantially increase the practical difficulty of doing so. In addition, some breeders may only require that within‐cohort selection accuracy be maximized. In which case, the conventional practice of evaluating and analyzing cohorts in separate trials should be followed. However, if gain from selection over time is desired, breeders should be concerned about the accuracy of parent selection, as this will affect the rate of genetic gain realized. When parent selection is of interest, analyzing multi‐environment, multi‐year data on multiple cohorts together in a GS model becomes more important, and therefore CR should be considered.

Some breeders have expressed concerns about CR that this simulation did not examine. First, CR could increase experimental error because preliminary cohorts may have taller height or lower lodging tolerance, thereby affecting neighboring plots. While this may be possible, in our experience with elite wheat germplasm the phenotypic differences between different cohorts are not that extreme. Certainly, the potential for this problem will be germplasm dependent. Another common concern about CR is that block sizes will be large within trials if all cohorts are randomized together, and therefore blocking will be less effective at controlling experimental error. This issue is easily solved by simply adding more incomplete blocks to the trial and, if necessary, additional design checks. Design checks can provide connectivity across incomplete blocks regardless of the number of blocks included, allowing for flexible expansion of experimental capacity.

While this study points out the benefits of CR in breeding programs, it is limited in scope. More comprehensive research examining CR and RR in breeding programs considering different germplasm, crops, and design details is warranted to understand to what degree our results can be generalized more broadly. Furthermore, our simulations were conservative and even provided potential competitive advantages to RR over CR to ensure experimental integrity and unbiased assessment for considering CR: (1) area main effects were not simulated, which would be well‐determined in CR but confounded with genetic effects under RR's trial‐restricted cohort allocation; (2) error variance was calculated per designated heritability level at the location level rather than area level to avoid area‐specific heterogeneity arising from differential genetic composition across RR areas; and (3) RR required the use of design checks to allow for genetic connectivity between cohort‐restricted areas; and utilized a larger number of blocks that inherently increased check number, resulting in a larger check composition that could potentially improve estimation of non‐genetic effects relative to CR. Further, we did not consider the potential for better resource allocation with CR relative to RR due to the reduced need for design check plots. This study also does not examine the relative merits of CR and RR when cohorts are more genetically distinct than those of this study. (We expect an even greater advantage from implementing CR when genetic differences between cohorts are great.) Nevertheless, this research exposes the need to challenge conventional thinking about how breeding trials are designed now that breeding has shifted from an art form to predictive science.

## AUTHOR CONTRIBUTIONS

**Arlyn Ackerman**: Data curation; formal analysis; investigation; methodology; software; validation; visualization; writing—original draft; writing—review and editing. **Jessica Rutkoski**: Conceptualization; data curation; formal analysis; investigation; methodology; resources; supervision; validation; writing—original draft; writing—review and editing.

## CONFLICT OF INTEREST STATEMENT

The authors declare no conflicts of interest.

## Data Availability

Simulation code and example datasets are publicly available at https://github.com/ackermanar/breeding‐cohort‐randomization. The repository includes R scripts for breeding simulation, statistical analysis workflows, result files, and documentation for replicating the study's core findings.

## References

[tpg270218-bib-0001] Atanda, S. A. , Govindan, V. , Singh, R. , Robbins, K. R. , Crossa, J. , & Bentley, A. R. (2022). Sparse testing using genomic prediction improves selection for breeding targets in elite spring wheat. Theoretical and Applied Genetics, 135(6), 1939–1950. 10.1007/s00122-022-04085-0 35348821 PMC9205816

[tpg270218-bib-0002] Atanda, S. A. , Olsen, M. , Crossa, J. , Burgueño, J. , Rincent, R. , Dzidzienyo, D. , Beyene, Y. , Gowda, M. , Dreher, K. , Boddupalli, P. M. , Tongoona, P. , Danquah, E. Y. , Olaoye, G. , & Robbins, K. R. (2021). Scalable sparse testing genomic selection strategy for early yield testing stage. Frontiers in Plant Science, 12, 658978. 10.3389/fpls.2021.658978 34239521 PMC8259603

[tpg270218-bib-0003] Bassi, F. M. , Bentley, A. R. , Charmet, G. , Ortiz, R. , & Crossa, J. (2016). Breeding schemes for the implementation of genomic selection in wheat (*Triticum* spp.). Plant Science, 242, 23–36. 10.1016/j.plantsci.2015.08.021 26566822

[tpg270218-bib-0004] Belamkar, V. , Guttieri, M. J. , Hussain, W. , Jarquín, D. , El‐basyoni, I. , Poland, J. , Lorenz, A. J. , & Baenziger, P. S. (2018). Genomic selection in preliminary yield trials in a winter wheat breeding program. G3 Genes|Genomes|Genetics, 8(8), 2735–2747. 10.1534/g3.118.200415 29945967 PMC6071594

[tpg270218-bib-0005] Bernardo, R. (2010). Breeding for quantitative traits in plants (3rd ed.). Stemma Press.

[tpg270218-bib-0006] Butler, D. G. , Cullis, B. R. , Gilmour, A. R. , Gogel, B. J. , & Thompson, R. (2018). ASReml estimates variance components under a general linear mixed model by residual maximum likelihood (REML). VSN International Ltd.

[tpg270218-bib-0007] Chandra, S. (1994). Efficiency of check‐plot designs in unreplicated field trials. Theoretical and Applied Genetics, 88(5), 618–620. 10.1007/bf01240927 24186119

[tpg270218-bib-0008] Clarke, G. P. Y. , & Stefanova, K. (2011). Optimal design for early‐generation plant‐breeding trials with unreplicated or partially replicated test lines. Australian & New Zealand Journal of Statistics, 53, 461–480. 10.1111/j.1467-842x.2011.00642.x

[tpg270218-bib-0009] Cobb, J. N. , Juma, R. U. , Biswas, P. S. , Arbelaez, J. D. , Rutkoski, J. , Atlin, G. , Hagen, T. , Quinn, M. , & Ng, E. H. (2019). Enhancing the rate of genetic gain in public‐sector plant breeding programs: Lessons from the breeder's equation. Theoretical and Applied Genetics, 132(3), 627–645. 10.1007/s00122-019-03317-0 30824972 PMC6439161

[tpg270218-bib-0010] Combs, E. , & Bernardo, R. (2013). Accuracy of genomewide selection for different traits with constant population size, heritability, and number of markers. The Plant Genome, 6(1), plantgenome2012.11.0030. 10.3835/plantgenome2012.11.0030

[tpg270218-bib-0011] Covarrubias‐Pazaran, G. , Gebeyehu, Z. , Gemenet, D. , Werner, C. , Labroo, M. , Sirak, S. , Coaldrake, P. , Rabbi, I. , Kayondo, S. I. , Parkes, E. , Kanju, E. , Mbanjo, E. G. N. , Agbona, A. , Kulakow, P. , Quinn, M. , & Debaene, J. (2022). Breeding schemes: What are they, how to formalize them, and how to improve them? Frontiers in Plant Science, 12, 791859. 10.3389/fpls.2021.791859 35126417 PMC8813775

[tpg270218-bib-0012] Cullis, B. R. , Smith, A. B. , Cocks, N. A. , & Butler, D. G. (2020). The design of early‐stage plant breeding trials using genetic relatedness. Journal of Agricultural, Biological and Environmental Statistics, 25(4), 553–578. 10.1007/s13253-020-00403-5

[tpg270218-bib-0013] Cullis, B. R. , Smith, A. B. , & Coombes, N. E. (2006). On the design of early generation variety trials with correlated data. Journal of Agricultural, Biological, and Environmental Statistics, 11(4), 381. 10.1198/108571106X154443

[tpg270218-bib-0014] Endelman, J. B. (2011). Ridge regression and other kernels for genomic selection with R package rrBLUP. The Plant Genome, 4(3), 250. 10.3835/plantgenome2011.08.0024

[tpg270218-bib-0015] Fisher, R. A. (1926). The arrangement of field experiements. Journal of the Ministry of Agriculture, 33, 503–515.

[tpg270218-bib-0016] Gaire, R. , Brown‐Guedira, G. , Dong, Y. , Ohm, H. , & Mohammadi, M. (2021). Genome‐wide association studies for fusarium head blight resistance and its trade‐off with grain yield in soft red winter wheat. Plant Disease, 105(9), 2435–2444. 10.1094/pdis-06-20-1361-re 33560886

[tpg270218-bib-0017] Gaire, R. , De Arruda, M. P. , Mohammadi, M. , Brown‐Guedira, G. , Kolb, F. L. , & Rutkoski, J. (2022). Multi‐trait genomic selection can increase selection accuracy for deoxynivalenol accumulation resulting from fusarium head blight in wheat. The Plant Genome, 15, e20188. 10.1002/tpg2.20188 35043582 PMC12807082

[tpg270218-bib-0018] Gaynor, R. C. , Gorjanc, G. , Bentley, A. R. , Ober, E. S. , Howell, P. , Jackson, R. , Mackay, I. J. , & Hickey, J. M. (2017). A two‐part strategy for using genomic selection to develop inbred lines. Crop Science, 57(5), 2372–2386. 10.2135/cropsci2016.09.0742

[tpg270218-bib-0019] Gezan, S. , Murray, D. , Oliveira, A. A. , & Galli, G. (2022). ASRgenomics: An R package with complementary genomic function (R package version 1.1.3) [Computer software]. VSN International. https://cran.r-project.org/package=ASRgenomics

[tpg270218-bib-0020] Heffner, E. L. , Lorenz, A. J. , Jannink, J. , & Sorrells, M. E. (2010). Plant breeding with genomic selection: Gain per unit time and cost. Crop Science, 50(5), 1681–1690. 10.2135/cropsci2009.11.0662

[tpg270218-bib-0021] Jannink, J.‐L. , Lorenz, A. J. , & Iwata, H. (2010). Genomic selection in plant breeding: From theory to practice. Briefings in Functional Genomics, 9, 166–177. 10.1093/bfgp/elq001 20156985

[tpg270218-bib-0022] Lorenz, A. , & Nice, L. (2017). Training population design and resource allocation for genomic selection in plant breeding. In R. Varshney , M. Roorkiwal , & M. Sorrells (Eds.), Genomic selection for crop improvement (pp. 7–22). Springer. 10.1007/978-3-319-63170-7_2

[tpg270218-bib-0023] Lorenz, A. , & Smith, K. P. (2015). Adding genetically distant individuals to training populations reduces genomic prediction accuracy in Barley. Crop Science, 55, 2657–2667. 10.2135/cropsci2014.12.0827

[tpg270218-bib-0024] Lorenz, A. J. , Chao, S. , Asoro, F. G. , Heffner, E. L. , Hayashi, T. , Iwata, H. , Smith, K. P. , Sorrells, M. E. , & Jannink, J.‐L. (2011). Chapter two genomic selection in plant breeding knowledge and prospects. Advances in Agronomy, 110, 77–123. 10.1016/b978-0-12-385531-2.00002-5

[tpg270218-bib-0025] Martin, R. J. , Eccleston, J. A. , Chauhan, N. , & Chan, B. S. P. (2006). Some results on the design of field experiments for comparing unreplicated treatments. Journal of Agricultural, Biological, and Environmental Statistics, 11(4), 394. 10.1198/108571106X154489

[tpg270218-bib-0026] Moehring, J. , Williams, E. , & Piepho, H.‐P. (2014). Efficiency of augmented p‐rep designs in multi‐environmental trials. Theoretical and Applied Genetics, 127(5), 1049–1060. 10.1007/s00122-014-2278-y 24553963

[tpg270218-bib-0027] Piepho, H. P. , & Williams, E. R. (2006). A comparison of experimental designs for selection in breeding trials with nested treatment structure. Theoretical and Applied Genetics, 113(8), 1505–1513. 10.1007/s00122-006-0398-8 17028902

[tpg270218-bib-0028] Robbins, K. , Backlund, J. E. , & Schnelle, K. (2012). Spatial corrections of unreplicated trials using a two‐dimensional spline. Crop Science, 52, 1138–1144. 10.2135/cropsci2011.08.0417

[tpg270218-bib-0029] Rothbard, S. , Etheridge, J. C. , & Murray, E. J. (2024). A tutorial on applying the difference‐in‐differences method to health data. Current Epidemiology Reports, 11(2), 85–95. 10.1007/s40471-023-00327-x

[tpg270218-bib-0030] Smith, A. , Ganesalingam, A. , Lisle, C. , Kadkol, G. , Hobson, K. , & Cullis, B. (2021). Use of contemporary groups in the construction of multi‐environment trial datasets for selection in plant breeding programs. Frontiers in Plant Science, 11, 623586. 10.3389/fpls.2020.623586 33603761 PMC7884452

[tpg270218-bib-0031] Smith, A. B. , Stringer, J. K. , Wei, X. , & Cullis, B. R. (2007). Varietal selection for perennial crops where data relate to multiple harvests from a series of field trials. Euphytica, 157(1–2), 253–266. 10.1007/s10681-007-9418-2

[tpg270218-bib-0032] Venables, W. , & Ripley, B. (2002). Modern applied statistics with S (4th ed.). Springer. https://www.stats.ox.ac.uk/pub/MASS4/

[tpg270218-bib-0033] Verges, V. L. , & Van Sanford, D. A. (2020). Genomic selection at preliminary yield trial stage: Training population design to predict untested lines. Agronomy, 10(1), 60. 10.3390/agronomy10010060

[tpg270218-bib-0034] Yan, W. (2021). Estimation of the optimal number of replicates in crop variety trials. Frontiers in Plant Science, 11, 590762. 10.3389/fpls.2020.590762 33519847 PMC7838102

